# Why Individuals Do (Not) Use Contact Tracing Apps: A Health Belief Model Perspective on the German Corona-Warn-App

**DOI:** 10.3390/healthcare11040583

**Published:** 2023-02-15

**Authors:** David Harborth, Sebastian Pape, Lukas Tom McKenzie

**Affiliations:** Faculty of Economics and Business, Goethe University Frankfurt, 60326 Frankfurt am Main, Germany

**Keywords:** contact tracing apps, SARS-CoV-2, COVID-19, health belief model, user adoption of health-related technologies, quantitative user study

## Abstract

The World Health Organization declared the emergence of the novel coronavirus (SARS-CoV-2) in January 2020. To trace infection chains, Germany launched its smartphone contact tracing app, the “Corona-Warn-App” (CWA), in June 2020. In order to be successful as a tool for fighting the pandemic, a high adoption rate is required in the population. We analyse the respective factors influencing app adoption based on the health belief model (HBM) with a cross-sectional online study including 1752 participants from Germany. The study was conducted with a certified panel provider from the end of December 2020 to January 2021. This model is primarily known from evaluations of medical treatments, such as breast cancer screenings, but it was rarely applied in prior work for a health-related information system such as the CWA. Our results indicate that intrinsic and extrinsic motivation to use the CWA are the strongest drivers of app use. In contrast, technical barriers, privacy concerns and lower income are the main inhibitors. Our findings contribute to the literature on the adoption of contact tracing apps by questioning actual users and non-users of the CWA, and we provide valuable insights for policymakers regarding influences of adoption and potential user groups of disease prevention technologies in times of pandemics.

## 1. Introduction

Shortly after a cluster of pneumonia cases of unknown aetiology was detected in Wuhan (China) in late 2019, the World Health Organization (WHO) declared the emergence of the novel coronavirus (SARS-CoV-2/COVID-19) in January 2020 [1]. In an effort to trace coronavirus infection chains, Germany launched an open-source smartphone contact tracing app (“Corona-Warn-App”) in June 2020. The Corona-Warn-App (CWA) informs users who were exposed to a person later tested positive on the basis of Exposure Notification APIs [2]. The CWA works independently and, in particular, does not rely on the health offices, which has the advantage that it can warn people even if the health offices are overloaded. The app is under active development, and since our survey, new features such as information on COVID-19 incident rates, the possibility for check-ins and a vaccination certification and test management were added. For the CWA to be successful, a high proportion of the German population has to adopt the technology and register themselves once they have been declared infected with COVID-19. Besides heavy advertisements from the German government, the development of the CWA in Germany was accompanied by public discussions about its usefulness, i.e., the claim that more than 50% of the population needed to use it to be effective [3], and data privacy issues. The discussion on data privacy issues started with the question of whether the CWA should facilitate a centralised or decentralised architecture [4]. A decentralised approach was chosen, resulting in ongoing claims by politicians that privacy would hinder a beneficial use of the CWA [5] while privacy experts pointed out that data protection is not a hindrance and necessary for a high adoption rate [6]. However, a high press coverage of these discussions could alter individuals’ perceptions, who usually are neither privacy experts nor epidemiologists [7,8]. On that ground, we investigate which variables have led German citizens to use or not use the CWA. We address this question with the theoretical lens of the Health Belief Model (HBM), which was originally developed in the 1950s and remains one of the most widely applied conceptual frameworks for health-related behaviours, especially disease prevention behaviours [9]. The HBM represents a profound and established model for analysing subjective expectations and barriers regarding the disease prevention behaviour (use of CWA) which is a function of the subjective value of an outcome as well as the subjective probability or expectation, that a particular action will achieve that outcome. However, since the original HBM did not consider infectious diseases, we had to adapt several constructs.

While the HBM has been applied to COVID-Tracing apps in earlier studies [10,11], both of them were conducted before the actual introduction of an app and worked by describing potential properties of the upcoming app and asking for the behavioural intention to use it. In contrast, our participants were sampled by users and non-users of the already existing CWA in Germany. We conducted a representative user study with the help of a market research institute with 1752 participants in Germany. We sampled participants evenly distributed into these two groups (users of the app, N = 896 and non-users, N = 856). Thus, we could utilise the actual use as the dependent variable. Furthermore, it has been shown that, in particular, the older population and those in lower-income households might also face technical barriers to using the app [12], which could not be considered in the previous HBM studies. With our study design, we can investigate such factors in a more realistic way. We investigate the effect of each (partially adapted) factor from the HBM on the likelihood of using the CWA based on a logistic regression model. Our results contribute to the theoretical IS knowledge base by establishing that the HBM is a useful theory for health information systems and can also be used to explain the behaviour of people for infectious diseases. Our results also contribute to the literature on contact tracing app adoption by showing that perceptions of technical barriers and privacy concerns, as well as a lower income, are the main inhibitors for adopting the CWA, while the intrinsic and extrinsic motivations to use the app are the main drivers.

## 2. The Health Belief Model

The health belief model was developed through a set of independently investigated research questions. It has been formulated in order to explain the widespread failure of people to participate in case-finding programs to prevent and detect asymptomatic diseases [9]. The design and dissemination followed the work by a group of behavioural psychologists in the U.S. Public Health Service between 1950 and 1960 at a time when health professionals were alarmed by the fact that few people were getting screened for tuberculosis (TB). The HBM states that health-related behaviours reflect both an individual’s level of fear of perceived health threats and the expected fear-reduction potential of taking a recommended action [13]. Based on this notion, we will examine how the perceived health threat of contracting SARS-CoV-2 will affect a person’s willingness to use the CWA. Originally, the HBM contained five theoretical constructs, which all depict the psychological explanation of whether and why people will take action to engage in disease preventives [13,14]. These constructs are (1) perceived susceptibility (perceptions of the likelihood of getting a disease or condition), (2) perceived severity (beliefs about the seriousness of the illness, condition, or unwanted outcome and the potential consequences), (3) perceived barriers (beliefs about the influences that discourage adoption of the promoted action or new behaviour), (4) perceived benefits (beliefs about the positive consequences of adopting the promoted action) and finally (5) cues to action (internal or external factors that could trigger health behaviour). The seminal work on operationalisations for these constructs by Champion (1984) [15] provides the basis for our research.

Medical studies have been using HBM for decades in order to measure constructs’ association with diseases and health behaviours, such as breast cancer [16,17,18], or vaccine adoption [19,20]. For example, studies on breast cancer find that a person’s adherence to mammography is significantly associated with greater perceived susceptibility, lower barriers, higher benefits, and cues in the form of recommendations from healthcare providers [21].

The HBM has also been applied to SARS-CoV-2 to explore adherence to precautionary measures and preventive behaviours (e.g., face mask-wearing, social distancing) in several countries, such as Ethiopia [22] or China [23]. Other studies analyse the acceptance of SARS-CoV-2 vaccines in various populations, such as Malaysia [24]. Important predictors of a definite intention to take a vaccine are high perceived benefits and lower perceived barriers to receiving the vaccine, and higher levels of perceived susceptibility to infection [22]. An important contribution of our study is the explicit focus on the form of the disease, namely an infectious disease, for with such a disease, not only the person’s own behaviour and health matters but also the behaviours and interests of others. Covering this aspect is one of the key parts of adapting and developing HBM constructs and hypotheses in the latter parts of this paper.

### HBM in the Context of Contact Tracing Apps

Prior work on the HBM in the context of contact tracing apps can be divided into two groups. The first group of work employs HBM-based items in their survey but did not further examine the actual influence of these constructs on use [25]. The second group of research uses similar constructs to those in the HBM but does not explicitly refer to the HBM to evaluate the reasons for using the CWA in Germany [26,27]. For example, Munzert et al. (2021) find an increased risk of severe illness, which is relatable to the construct of perceived severity in the HBM, to encourage a higher uptake rate of CWA. To the best of our knowledge, there are only two studies which use the HBM in the context of tracing apps while focusing on a specific country, such as Belgium [10] or France [11]. However, in contrast to our study, which explains user behaviour, these studies focus on intentions or potential use. Several articles in the IS literature show that there is a so-called intention-behaviour gap which might introduce biases in the results of such studies, especially when dealing with privacy-related judgements [28,29]. Thus, by having a dependent variable based on quotas, our analysis advances the results of prior work as we can significantly reduce the bias due to self-reported information and social desirability bias.

## 3. Research Hypotheses

Against the backdrop of the related work and prior literature on contact tracing apps (see Section 2), we introduce our adapted HBM for the case of the German CWA in this section. We needed to adjust the model at several points in order to address that we are dealing with an infectious disease which also has immediate medical consequences on other people (unlike, for example, cancer as in the original operationalisation by Champion (1984)). This led to several changes in the constructs themselves as well as in the items of the constructs (see Appendix A for the respective items). We will discuss them in the following subsections and derive the respective hypotheses based on the HBM.

### 3.1. Perceived Susceptibility

Perceived susceptibility is taken to a large extent from Champion (1984), whereas we left out three of the original six items and replaced them with two items reflecting the infectious aspect of the disease covering the likelihood of getting infected on the job and, more generally, in daily life. Following the notion of the HBM, individuals are more likely to use disease prevention tools such as the CWA if they think that there is a high likelihood of becoming infected with SARS-CoV-2. Thus, we hypothesise:

**Hypothesis (H1).**  The perceived susceptibility (PS) of getting SARS-CoV−2 has a positive impact on the likelihood of using the CWA.

### 3.2. Perceived Severity

Perceived severity was originally operationalised as a 12-item construct which is appropriate for diseases such as cancer (Champion 1984). We decided to split it into three distinct dimensions to cover the specifics of an infectious disease which can also harm others and can have certain social effects on social relationships and the career. Thus, we evaluate the perceived medical consequences on oneself (corresponding to the analysis of other non-infectious diseases investigated in the literature with the HBM), the perceived severity of the medical consequences when one becomes infected and endangers others, such as family members, friends and work colleagues and the perceived social consequences (non-medical) that come along with infection with SARS-CoV-2, including financial and career aspects as well as risks to social relationships [30]. The respective items for medical consequences on oneself and related to social consequences are taken from Champion (1984), except for the third item of perceived severity related to social consequences, which we added to account for social relationships. The construct of perceived severity of medical consequences on others is self-made. The different dimensions of severity represent a threat for individuals which should, according to the HBM, cause them to engage in disease prevention, i.e., increase the likelihood of using the CWA, leading to the following hypotheses:

**Hypothesis (H2).**  The perceived severity of medical consequences (PMC) on oneself when getting SARS-CoV-2 has a positive impact on the likelihood of using the CWA.

**Hypothesis (H3).**  The perceived severity of medical consequences on others (PSO) by getting SARS-CoV-2 has a positive impact on the likelihood of using the CWA.

**Hypothesis (H4).**  The perceived severity of social consequences (PSC) when getting SARS-CoV-2 has a positive impact on the likelihood of using the CWA.

### 3.3. Perceived Benefits

We changed the construct perceived benefits compared to the original construct as we needed to adapt it to disease prevention with its specific characteristics from doing self-breast exams (Champion 1984) to using the CWA. According to the HBM, the individual’s beliefs regarding the effectiveness and perceived benefits of reducing the disease threat are important factors influencing the use of disease preventives. Thus, we hypothesise:

**Hypothesis (H5).**  The perceived benefits (PB) of using the CWA have a positive impact on the likelihood of using the CWA.

### 3.4. Perceived Barriers

Perceived barriers are described as the potential negative aspects of a particular health action which impede the implementation of disease preventives. A cost-benefit analysis occurs wherein an individual weighs the action’s expected benefits with perceived barriers. Thus, it can be stated that “[…] combined levels of susceptibility and severity provide the energy or force to act and the perception of benefits (minus barriers) provide a preferred path of action” [9]. We differentiate these barriers into two types covering the technical knowledge and understanding of the app and privacy concerns. We relied on two new constructs operationalising these dimensions as they are very specific to the health action. On the one hand, there were several reports of technical difficulties surrounding the introduction of the app in Germany (e.g., not having a suitable smartphone or operation system) [31]. Thus, we include a self-made construct covering this aspect. On the other hand, privacy concerns were heavily discussed prior to the introduction of the CWA in Germany [32]. In addition, prior work shows the importance of privacy in the adoption of contact tracing apps (see Section 2). Thus, we included this construct as well. The items for this construct are adapted from prior work [33,34]. Thus, the resulting hypotheses are as follows:

**Hypothesis (H6).**  The perceived lack of technical knowledge and understanding (PTB) of the CWA has a negative impact on the likelihood of using the CWA.

**Hypothesis (H7).**  The perceived privacy concerns (PC) related to the CWA have a negative impact on the likelihood of using the CWA.

### 3.5. Cues to Action

An internal or external motivational drive is necessary to produce health-promoting behaviours. The intensity of motivation needed to trigger the decision-making process to use the CWA is another important factor in the HBM next to perceived susceptibility, severity, benefits, and barriers [9]. As cues to action were, to the best of our knowledge, not operationalised before for the case of infectious disease, we had to develop it ourselves. We split cues to action (named “motivation” by Champion (1984)) into two separate constructs to be able to differentiate the potentially different impacts of intrinsic motivation and extrinsic motivation on individual behaviour [35]. Intrinsic motivation reflects an individual’s inner drive to use the CWA to combat the disease (including registering positive test results and regularly checking for risk encounters). In contrast, extrinsic motivations are drivers coming from external events such as infections of close relatives, the overall number of infections in Germany and the world, as well as incentives for the social life or work. The resulting hypotheses are as follows:

**Hypothesis (H8).**  The intrinsic motivation (IM) to avoid COVID-19 infections for oneself or others has a positive impact on the likelihood of using the CWA.

**Hypothesis (H9).**  The extrinsic motivation (EM) to avoid COVID-19 infections for oneself or others has a positive impact on the likelihood of using the CWA.

## 4. Methodology

Based on the adapted HBM and our research hypotheses, we develop the research model and describe the data collection and processing in the section.

### 4.1. Research Model

Based on our research hypotheses derived from the HBM, we develop the following logistic regression model to assess the likelihood of an individual using the CWA based on the dependent observable variable CWA_USE_ (user of CWA = 1, non-user = 0). We also include several control variables in order to address the issue of endogeneity due to omitted variables. Thus, we include age, gender, education, income, smartphone experience and the health condition of participants (whether they themselves or family/close relatives belong to a COVID-19 risk group (Equation (1)).

(1) $$CWA_{USE}=\beta_{0}+\beta_{1}PS+\beta_{2}PMC+\beta_{3}PSO+\beta_{4}PSC+\beta_{5}PB+\beta_{6}PTB+\beta_{7}PC+\beta_{8}IM+\beta_{9}EM+\beta_{j}Control_{j}$$

### 4.2. Data Collection

We considered the ethical issues of the user study before collecting the data. The ethics board of the authors’ university provides an extensive checklist, and our study was qualified as exempt from an ethics review and therefore approved. We conducted the online study with a certified panel provider in Germany from the end of December 2020 to January 2021 (the panel provider is certified following the ISO 20252 norm). The survey was programmed with the survey software LimeSurvey (version 2.72.6) [36] and hosted on a university server. We sampled the participants in a way to achieve a representative sample for Germany. For that purpose, we set quotas to end up with approximately 50% females and 50% males in the sample as well as a distribution of age following the EUROSTAT2018 census [37]. Furthermore, we set a quota to end up with half of the sample using the CWA and the other half not using it. This quota serves as the dependent variable for our later logistic regression analysis.

## 5. Results

First, we descriptively discuss our sample and the factors of the HBM. Following this analysis, we discuss the results of the binomial logistic regression. All analyses were conducted in R (version 4.0.3).

### 5.1. Descriptive Analysis

Our sample consists of 1752 participants. Following EUROSTAT 2018, participants are representative of the German population with respect to age and gender. The same diversity can be observed for income and education (Table 1). Some 896 participants use the CWA (51.14%), and 856 do not (48.86%): 1299 use Android (74.14%), 436 use iOS (24.89%) and 17 stated to use other mobile operating systems (0.97%). This distribution of operating systems is representative for Germany [38].

Since we deliberately divided the sample into two approximately equal groups (CWA users and non-users), we need to check for statistically significant differences in the demographics between these groups. This is required in order to rule out the confounding influences of these variables on our results. We conducted a Shapiro–Wilk test for normality for all variables, and the variables are all not normally distributed. Thus, we conducted Wilcoxon rank-sum tests to analyse whether there are significant differences between CWA users and non-users. Age and gender show no differences since we deliberately sampled our participants with equal distributions with respect to age and gender. There are statistically significant differences between users and non-users of the CWA for the remaining demographics. The income is statistically significantly higher for the users compared to the non-users. However, the median is the same, which is why we argue that the absolute difference is not having a substantial confounding effect on our later analysis. The same argumentation holds for education, with a median of 4 for users and 3.5 for non-users and smartphone experience in years, with a mean 8.77 for users and 8.35 for non-users. We also calculate descriptive statistics for the used constructs. The means and standard deviations are shown in Table 2. As we measured the answers on a 7-point Likert scale, values equal to 4 indicate that participants neither agree nor disagree with the statements. We can see that the mean values for all variables from the HBM are statistically significantly different between users and non-users of the CWA, except for the construct perceived severity related to social consequences. All variables of the HBM have values and differences, as one would expect between the groups.

Susceptibility and the medical severity dimensions (on oneself and others), and thereby, the overall judgement on the potential threat of SARS-CoV-2, are significantly higher for users of the CWA. However, it must be noted that the perceived likelihood of getting SARS-CoV-2 (susceptibility) is not perceived as pertinent to the users of the CWA as well. In contrast, the severity related to medical consequences on oneself and on others is assessed as more critical by users, whereas non-users are indifferent. A clearer difference between the groups can be seen in the benefits of the CWA (users tend to agree that it has benefits and non-users rather disagree), the privacy concerns and the intrinsic motivation. Both groups tend to disagree that there are technical barriers and that they are extrinsically motivated (although statistically significantly different). In order to assess the reliability of the constructs, we calculated polychoric correlations between the items of the respective constructs and used this matrix to conduct a factor analysis. Polychloric correlations are usually used for categorical variables [39]. The loadings for each item of the constructs are at least 0.5, with an explained variance of at least 50.3% or higher. Values for Cronbach’s Alpha are shown in Table 2. Thus, the reliability of the constructs is established, and we can use the variables as they are for the regression analysis.

### 5.2. Regression Analysis

We conduct the logistic regression analysis in the following way. First, based on our finding that the constructs are valid and reliable, we calculate the mean sum scores of the constructs in order to have a single construct which is usable in the regression analyses. Second, we calculate the base model only, including the constructs of the HBM. Third, we calculated the full model with demographics with a sample size of 1571 as we needed to exclude participants who preferred not to provide their gender or income. The results of the regression analyses are shown in Table 3.

In order to evaluate and discuss the results of the logistic regression model, we first derive a reduced model (see Equation (2)) with all statistically significant variables and estimate whether the full model explains the dependent variable better than the reduced one based on the likelihood ratio test. We have to reject this hypothesis which indicates that the reduced model, and consequently all variables shown as significant, explain the use of the CWA with the same or better accuracy. In line with these results, the Bayesian information criterion (BIC), as well as the Akaike information criterion (AIC), are lower for the reduced model compared to the full model (BIC_reduced_ = 1096.611 versus BIC_full_ = 1176.289/AIC_reduced_ = 1032.298 versus AIC_full_ = 1036.943).

(2) $$CWA_{USE}=\beta_{0}^{r}+\beta_{1}^{r}PS+\beta_{2}^{r}PSO+\beta_{3}^{r}PB+\beta_{4}^{r}PTB+\beta_{5}^{r}PC+\beta_{6}^{r}IM+\beta_{7}^{r}EM+\beta_{8}^{r}Income$$

**Figure A1:**
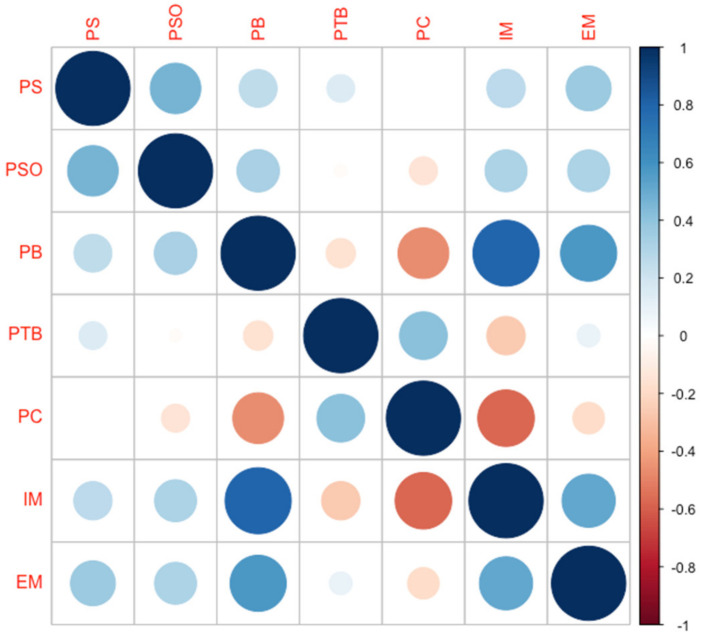
Pairwise correlations between the latent independent variables.

As we conduct a logistic regression analysis, we need to evaluate overdispersion, which can happen if there is more variance in the data in comparison to the assumed distribution. We check for overdispersion by calculating the ratio of the residual deviance and the residual degrees of freedom of the logistic model. Literature suggests that the ratio should not be considerably larger than 1 [40]. The ratio in our case is 0.647, indicating that overdispersion is not an issue. As there is no such metric as R^2^ for logistic regressions, we use McFadden’s R^2^ as a pseudo R^2^ measure. McFadden’s R^2^ ranges from 0 to 1 with values from 0.2 to 0.4, indicating an “excellent fit” [41]. In our case, the reduced model has a McFadden’s R^2^ equal to 0.536, indicating an excellent predictive power of the independent variables. We additionally conducted two tests to assess the statistical significance and relevance of each independent variable. The Wald test indicates that all variables in the reduced model contribute to the explanatory power of the model (*p* < 0.05 for all variables of the reduced model). Furthermore, we assessed the relative importance based on the t-values of the variables, which also confirms that all variables in the reduced model are significant (t-values > 1.96 for all variables of the reduced model). Finally, we evaluate multicollinearity, which occurs when independent variables are correlated with each other. We plot the pairwise correlations and find that intrinsic motivation and perceived benefits might pose a problem regarding collinearity (see Figure A1, Appendix B). We will consider the potential effect of this correlation in the discussion of the results.

In order to interpret the statistically significant coefficients, we calculate the average marginal effects for the reduced model, as the coefficients themselves are not interpretable as they are (see Table 4). We refer to the marginal effects in the following discussion as they can be measured in percentage points and can be interpreted in isolation.

We find that participants with a higher perceived susceptibility, higher perceptions with respect to the benefits of the CWA as well as a higher intrinsic and extrinsic motivation, have an increased probability of using the CWA. We find the lowest marginal effect of 0.017 for perceived susceptibility, corresponding to a 1.7 percentage point increase. The highest marginal effect can be observed for intrinsic motivation, with a one-unit increase in it corresponding to a 7.4 percentage points increase in the probability of using the CWA. The variables which have a negative effect on the probability of using the CWA are the perceived severity of others, perceived technical barriers, privacy concerns, and income. The largest negative effects can be observed for perceived technical barriers (6.3 percentage point decrease in the probability of using the CWA) and privacy concerns (3.5 percentage point decrease in the probability of using the CWA).

Interestingly, the hypotheses for none of the other threat dimensions of the HBM (severity of medical consequences on oneself, others and social consequences) can be confirmed. We even have to reject H3 (positive effect of perceived severity on others) as the marginal effect indicates that a one-unit increase in this variable decreases the probability of using the CWA by 2 percentage points if participants feel that infection with the virus harms others. This is interesting as the concept of warning others in the case of an infection is the key function of a contact tracing app.

## 6. Discussion

Our results indicate that the use of the German CWA app is mainly influenced by five variables. The likelihood of using the CWA is positively influenced by participants’ intrinsic motivation and extrinsic motivation. Interestingly, several variables representing threat dimensions of SARS-CoV-2 are not significant (e.g., perceived medical consequences) or have relatively small effect sizes (perceived susceptibility). Perceived severity on others has a significant (small) negative effect. The latter finding is surprising since the mean of perceived severity on others is significantly larger for CWA-Users than for Non-CWA-Users (cf. Table 2). Further research will be required to determine if it is an artefact of the regression or indicates a serious effect. The general understanding of participants that infections of others are a reason for using the CWA can be seen in the positive effect of extrinsic motivation on use (although it has to be noted that participants tend to disagree in both groups to items of this construct (see Table 2)).

The three other variables with the largest effects are perceived technical barriers, privacy concerns and income. This indicates that there is a relatively high intrinsic motivation with a large effect on app use on the one hand (also visible by the large mean of 5.77 in the group of users), but this factor is attenuated by technical barriers and a lack of understanding (e.g., explanations missing, compatibility with a smartphone not given) and privacy concerns. In addition, our results indicate that socioeconomic factors might influence app use as a lower income tends to decrease the likelihood of using the app in our sample, whereas a higher level of education increases the likelihood. These findings partially correspond to prior work showing that the older population and those in lower-income households might also face technical barriers to using the app, e.g., by having limited smartphone access or an old smartphone [12]. This finding is also discussed by other researchers pointing towards the larger theme of increasing inequality during the pandemic as citizens who do not have the respective resources or who do not want to engage in certain behaviours (e.g., because of missing information about the technology or the disease) are basically shut out from employing all possible resources to protect themselves and others [42]. In the context of demographic influences, it is interesting that neither age nor gender has a significant effect. The same holds for the variables indicating whether participants themselves or close relatives and family members belong to a SARS-CoV-2 risk group. For the risk group variables, the same argumentation could be made for perceived severity on others, namely that participants do not perceive a contract tracing app as a necessary disease prevention tool in their inner social circles. 

These results are important for policymakers as we heard many discussions in Germany which were based on the idea that there could be a kind of trade-off between a “proper” contact tracing app and other measures such as curfews, lockdowns or social distancing. It was often argued that a contact tracing app with less focus on privacy and more functionalities could enable more freedom [5]. However, our results indicate that this idea is highly questionable not only because of the simple fact that less privacy (if there is anything like this) does not cause the app to function better [6]. The results even indicate that greater privacy concerns could negatively impact the likelihood of the adoption of the CWA.

All these findings indicate that people are mainly driven by an inner motivational drive and responsibility to fight the disease, which could only be harmed by high barriers in the form of a more intrusive app collecting unnecessary amounts of data or technical barriers such as compatibility issues.

### Limitations and Future Work

This study has certain limitations. First, although we work with an observed dependent variable, the independent variables are self-reported, which could cause biases (e.g., due to the social desirability bias). Second, due to the contextual change of considering an infectious disease in the HBM, we had to adapt existing items (e.g., perceived benefits) and develop new items (e.g., extrinsic motivation). These are not validated, and thus do not have the same maturity as the original items in the well-established HBM and may be less reliable. However, in order to be able to report recent results about a COVID-19-related topic, we decided against validating the new items first. Third, the analysis did not include interaction effects between variables. However, we plan to conduct a second wave of the survey in order to conduct a longitudinal analysis of the changes in user behaviour of the CWA along with changing infection numbers. In the consequent analysis, interaction effects between HBM factors and demographics will be investigated in the context of a larger study. This is necessary as our study indicates a certain socio-demographic influence on the use of contact tracing apps. An analysis of interaction effects between the factors of the HBM and variables reflecting the social status of individuals (such as education or income) is a highly interesting avenue for future work as it addresses the question: to what extent can policymakers expect certain social groups to participate in pandemic-fighting tools? Such findings from IS research could even inform research on herd immunity in the context of vaccinations, which requires the majority of a society to participate in order to come back to normality. Lastly, every country had very specific types of contact tracing apps with accompanying political and social discussions. These specific features and discussions shape the perceptions of individuals in the respective countries, causing a limited generalisability of our results for contact tracing apps in other countries.

## 7. Conclusions

We conducted a representative user study with 1752 participants in Germany on the use of the German contact tracing app (Corona-Warn-App). We sampled participants into two groups (users of the app, N = 896 and non-users, N = 856). We base our research on the health belief model, which explains why individuals engage in disease prevention behaviours. We adapted the model which was originally applied in the medical context (e.g., mammography for breast cancer prevention) to infectious disease and the prevention behaviour of using a contact tracing app.

We find that the probability of using the CWA is mainly positively influenced by the intrinsic and extrinsic motivation of individuals and negatively influenced by perceived technical barriers, privacy concerns, and a lower income. We contribute to the current stream of literature on the adoption of contact tracing apps, disease prevention tools in general, and the use of the HBM in the context of technologies by introducing an adapted HBM suited to investigate infectious diseases and new information systems to cope with the associated challenges of those diseases. Furthermore, we argue that our results from the IS domain can contribute to a better understanding of users and, in particular, suggest that the perceived technical barrier should be reduced, e.g., by providing further elucidations. It has to be determined in future work whether the results can be transferred to other behaviours or use of measures in the pandemic (e.g., the willingness to get a vaccination or to obey rules in the pandemic for reducing contact and hygiene) and which factors would foster an environment for generating understanding of and participation in the measures.

## Figures and Tables

**Table 1:** Sample Demographics († 5 GCSEs at Grade C and above).

Demographics	N	%	Demographics	N	%
Age			Gender		
18–29 years	371	21.17%	Female	894	51.03%
30–39 years	316	18.04%	Male	853	48.69%
40–49 years	329	18.78%	Diverse	4	0.23%
50–59 years	431	24.60%	Prefer not to say	1	0.05%
60 years and older	305	17.41%	Education		
Net income			No degree	8	0.46%
500€−1000€	160	9.13%	Secondary school	187	10.67%
1000€−2000€	402	22.95%	Secondary school †	574	32.76%
2000€−3000€	404	23.06%	A levels	430	24.54%
3000€−4000€	314	17.92%	Bachelor’s degree	240	13.70%
More than 4000€	292	16.67%	Master’s degree	285	16.27%
Prefer not to say	180	10.27%	Doctorate	28	1.60%

**Table 2:** Descriptive Statistics for the Constructs.

	CWA	0	1	*p*-Value Significance of Difference
Variable (Cronbach’s ∝)		N = 856 (Mean, sd)	N = 896 (Mean, sd)	
Perceived Susceptibility (∝=0.79)		3.40 (1.35)	3.81 (1.31)	<0.001
Perceived Medical Consequences (∝=0.87)		3.86 (1.49)	4.49 (1.17)	<0.001
Perceived Severity on Others (∝=0.83)		4.32 (1.64)	4.92 (1.42)	<0.001
Perceived Social Consequences (∝=0.74)		3.04 (1.53)	3.12 (1.52)	0.308
Perceived Benefits (∝=0.90)		3.05 (1.35)	4.72 (1.22)	<0.001
Perceived Technical Barriers (∝=0.88)		2.74 (1.41)	1.60 (1.07)	<0.001
Privacy Concerns (∝=0.96)		4.64 (1.70)	2.57 (1.52)	<0.001
Intrinsic Motivation (∝=0.89)		3.25 (1.68)	5.97 (1.18)	<0.001
Extrinsic Motivation (∝=0.85)		2.33 (1.27)	3.41 (1.45)	<0.001

**Table 3:** Logistic Regression Model on Using the CWA (*** *p* < 0.001, ** *p* < 0.01, * *p* < 0.05).

	(1) Base Model (N = 1752)	(2) Full Model (N = 1571)
**DV**: Are you using the Corona-Warn-App on your smartphone? (y/n)		
Perceived Susceptibility	0.177 * (2.261)	0.176 * (2.012)
Perceived Medical Consequences	−0.080 (−1.023)	0.011 (0.124)
Perceived Severity on Others	−0.163 ** (−2.636)	−0.227 *** (−3.301)
Perceived Social Consequences	0.052 (0.859)	0.058 (0.899)
Perceived Benefits	0.210 ** (2.650)	0.218 * (2.485)
Perceived Technical Barriers	−0.631 *** (−9.467)	−0.637 *** (−8.693)
Privacy Concerns	−0.329 *** (−6.497)	−0.358 *** (−6.457)
Intrinsic Motivation	0.749 *** (11.029)	0.775 *** (10.333)
Extrinsic Motivation	0.416 *** (5.767)	0.433 *** (5.409)
Income 5 (€500 to €1000)		−0.831 ** (−2.404)
Age, gender, education, smartphone experience, SARS-CoV-2 risk group (participant herself or family/close relatives)		**Not significant**

**Table 4:** Marginal effects (average partial effects) of the independent variables of the reduced model.

DV: CWA Use (y/n)	Marginal Effect
Perceived Susceptibility	0.017
Perceived Severity on Others	−0.020
Perceived Benefits	0.021
Perceived Technical Barriers	−0.063
Privacy Concerns	−0.035
Intrinsic Motivation	0.074
Extrinsic Motivation	0.047
Income 5 (€500 to €1000)	−0.098

## Data Availability

The data presented in this study are available on request from the corresponding author.

## Appendix A

All items were measured on a 7-point Likert scale and adapted from Champion (1984), if not otherwise indicated.

1. Perceived susceptibility (PS)

PS1. My chances of being infected by COVID-19 are great.
PS2. My physical health makes it more likely that an infection by COVID-19 will have serious consequences.
PS3. I worry a lot about being infected by COVID-19.
PS4. My job involves a high risk to be infected by COVID-19.
PS5. My daily life involves a high risk to be infected by COVID-19.

2. Perceived severity related to medical consequences on oneself (PMC)

PMC1. The thought of COVID-19 scares me.
PMC2. COVID-19 is a hopeless illness.
PMC3. Health issues I would experience from COVID-19 would last a long time.
PMC4. If I got infected by COVID-19, it would be more serious than other diseases.
PMC5. If I had COVID-19 my whole life would change.

3. Perceived severity on others (PSO) (self-made)

PSO1. The health of my friends would be at risk if I became infected with COVID-19.
PSO2. My family’s health would be at risk if I became infected with COVID-19.
PSO3. The health of my work colleagues would be at risk if I became infected with COVID-19.
PSO4. The health of other contacts would be at risk if I became infected with COVID-19.

4. Perceived severity related to social consequences (PSC)

PSC1. My financial security would be at risk if I became infected with COVID-19.
PSC2. If I became infected with COVID-19, my career would be at risk.
PSC3. My social relationships (family, friends) would be at risk if I became infected with COVID-19.

5. Perceived benefits (PB)

PB1. Using the Corona-Warn-App makes me feel safer.
PB2. I have a lot to gain by using the Corona-Warn-App.
PB3. The Corona-Warn-App can help me to identify contacts to infected individuals.
PB4. If I use the Corona-Warn-App I am able to warn others in case I am infected with COVID-19.
PB5. I feel that the usage of the Corona-Warn-App is beneficial to combat COVID-19.

6. Perceived technical barriers (PTB) (self-made)

PTB1. I am afraid to use the Corona-Warn-App because I don’t understand how it works.
PTB2. I don’t know how to go about using the Corona-Warn-App.
PTB3. Installing the Corona-Warn-App takes too much time.
PTB4. The installation of the Corona-Warn-App is associated with technical problems.

7. Perceived barriers related to privacy concerns (PC) [33]

PC1. I think the Corona-Warn-App gathers far too much of my personal information.
PC2. I worry that the Corona-Warn-App leaks my personal information to third-parties.
PC3. I am concerned that the Corona-Warn-App violates my privacy.
PC4. I am concerned that the Corona-Warn-App misuses my personal information.
PC5. I think that the Corona-Warn-App collects my location data.

8. Intrinsic motivation (IM) (self-made)

IM1. I feel responsible to register a positive test result into the Corona-Warn-App.
IM2. I feel responsible to use the Corona-Warn-App regularly to inform myself of potential risk encounters.

9. Extrinsic motivation (EM) (self-made)

EM1. Potential infections of family members or friends with COVID−19 affect my decision to use the Corona-Warn-App.
EM2. The number of COVID-19 infections in Germany affects my decision to use the Corona-Warn-App.
EM3. The number of COVID-19 infections in the world affects my decision to use the Corona-Warn-App.
EM4. Using the Corona-Warn-App has advantages for my social life.
EM5. The use of the Corona-Warn-App is requested for my work.

**Control Variables**

Age, gender, education, income, smartphone experience, Oneself or close relatives/family COVID-19 risk group (y/n)
